# A genetic approach to study the relationship between maternal Vitamin D status and newborn anthropometry measurements: the Vitamin D pregnant mother (VDPM) cohort study

**DOI:** 10.1007/s40200-019-00480-5

**Published:** 2020-01-27

**Authors:** Arif Sabta Aji, Erwinda Erwinda, Rosfita Rasyid, Yusrawati Yusrawati, Safarina G Malik, Buthaina Alathari, Julie Anne Lovegrove, Nur Indrawaty Lipoeto, Karani Santhanakrishnan Vimaleswaran

**Affiliations:** 1 Department of Biomedical Science, Faculty of Medicine, Andalas University, Padang, 25127 Indonesia; 2 Department of Nutrition, Faculty of Health Sciences, Alma Ata University, Yogyakarta, 55183 Indonesia; 3 Department of Public Health, Faculty of Medicine, Andalas University, Padang, West Sumatra 25127 Indonesia; 4 Department of Obstetrics and Gynecology, Faculty of Medicine, Andalas University, Padang, 25127 Indonesia; 5 Eijkman Institute for Molecular Biology, Jakarta, 10430 Indonesia; 6 Hugh Sinclair Unit of Human Nutrition, Department of Food and Nutritional Sciences, University of Reading, Reading, UK; 7 Department of Nutrition, Faculty of Medicine, Andalas University, Padang, 25127 Indonesia

**Keywords:** Vitamin D, Single nucleotide polymorphisms, 25-hydroxyvitamin D, Pregnancy, Newborn anthropometry, Genetic risk score, West Sumatra

## Abstract

**Purpose:**

Adverse effects of maternal vitamin D deficiency have been linked to adverse pregnancy outcomes. We investigated the relationship between maternal vitamin D status and newborn anthropometry measurements using a genetic approach and examined the interaction between genetic variations in involved in vitamin D synthesis and metabolism and maternal vitamin D concentrations on newborn anthropometry.

**Methods:**

The study was conducted in 183 pregnant Indonesian Minangkabau women. Genetic risk scores (GRSs) were created using six vitamin D–related single nucleotide polymorphisms and their association with 25-hydroxyvitamin D [25(OH)D] levels and newborn anthropometry (183 infants) were investigated.

**Results:**

There was no significant association between maternal 25(OH)D concentrations and newborn anthropometry measurements (*P* > 0.05, for all comparisons). After correction for multiple testing using Bonferroni correction, GRS was significantly associated with 25(OH)D in the third trimester (*P* = 0.004). There was no association between GRS and newborn anthropometric measurements; however, there was an interaction between GRS and 25(OH)D on head circumference (*P* = 0.030), where mothers of neonates with head circumference < 35 cm had significantly lower 25(OH)D if they carried ≥4 risk alleles compared to those who carried ≤3 risk alleles.

**Conclusion:**

Our findings demonstrate the impact of vitamin D-related GRS on 25(OH)D and provides evidence for the effect of vitamin D-related GRS on newborn anthropometry through the influence of serum 25(OH)D levels among Indonesian pregnant women. Even though our study is a prospective cohort, before the implementation of vitamin D supplementation programs in Indonesia to prevent adverse pregnancy outcomes, further large studies are required to confirm our findings.

**Electronic supplementary material:**

The online version of this article (10.1007/s40200-019-00480-5) contains supplementary material, which is available to authorized users.

## Introduction

As one of the tropical countries in Southeast Asia located at the equator, Indonesia has an abundant sunlight all year round. According to recent studies, vitamin D deficiency in Indonesian women ranges between 60 and 95% [1–5]. Adequacy of maternal vitamin D status is important for the development of bone, teeth, immune system and general growth of the foetus [5]. Vitamin D insufficiency during pregnancy have been shown to be associated with adverse pregnancy outcomes such as small-for-gestational-age (SGA), neurodevelopment and cognitive impairment, high blood pressure in women and infants, respiratory infections, increased incidence of infants treated in neonatal intensive care unit, and health outcomes in infants such as asthma, atopic allergy, and autoimmune disorders such as type 1 diabetes mellitus [6–11].

Hereditary factors have been shown to affect 29% to 80% of serum 25-hydroxyvitamin D [25(OH)D] concentrations [11]. Candidate gene studies have identified twelve genes based on the genome-wide association studies (GWAS) for 25(OH)D *(GC, CYP24A1, CYP2R1, DHCR7)* [12], GWAS for skin colour/tanning (interferon regulatory factor 4 *(IRF4)*; melanocortin 1 receptor *(MC1R)*; oculocutaneous albinism type 2 *(OCA2)*; solute carrier family 45, member 2 *(SLC45A2)*; tyrosinase (oculocutaneous) *(TYR)*) [13–15], and candidate gene studies for vitamin D pathway genes *(VDR,* cytochrome P450, family 27, subfamily A, polypeptide 1 *(CYP27A1)*; cytochrome P450, family 27, subfamily B, polypeptide 1 *(CYP27B1))* [16]. Recent GWASs have confirmed the association of six genetic variants in the following genes *(*short/branched chain acyl-CoA dehydrogenase *(ACADSB), GC, DHCR7, CYP2R1, and CYP24A1)* with 25OHD levels [12, 17], and these variants were found near genes involved in cholesterol synthesis, hydroxylation, and vitamin D transport that affects vitamin D status. The metabolic pathways and synthesis of vitamin D are regulated by the specific genes present in the pathway and the pathway is initiated by the exposure to UVB rays (vitamin D_3_) and dietary intake of vitamin D sources (vitamin D_2_).

Previous GWASs [12, 17] have identified common genetic variations that influence vitamin D status in western populations; however, very few studies have investigated the influence of common genetic variations on vitamin D status in populations within Southeast Asia, especially in Indonesian population. In this study, we explored the association between maternal vitamin D status and newborn anthropometry measurements using a genetic approach. Given the high level of confounding factors that exists between maternal vitamin D status and newborn anthropometry measurements, we used genetic variants as markers of maternal vitamin D status and tested for their association with newborn anthropometry measurements as genetic associations are less prone to confounding. In addition, we also investigated whether the association between genetic variants and newborn anthropometry measurements were modified by 25(OH)D concentrations in Indonesian pregnant women from West Sumatra.

## Methodology

### Study population

The study was conducted among singleton pregnant women of West Sumatran Vitamin D Pregnant Mother (VDPM) cohort study in West Sumatra, from July 2017 to April 2018. The study was performed at community health centres in five cities (Padang, Pariaman, Payakumbuh, Padang Pariaman, and Lima Puluh Kota) in West Sumatra, Indonesia. In this study, participants were followed up from the first trimester (T1) to third trimester (T3) of pregnancy and at delivery to determine newborn anthropometry measurements (birth weight, birth length and head circumference). This study was conducted in accordance with the declaration of Helsinki and approved by the Ethics Committees of Medical Faculty, Andalas University (No. 262/KEP/FK/2016). All women provided written informed consent prior to the start of the data collection.

**Fig. 1:**
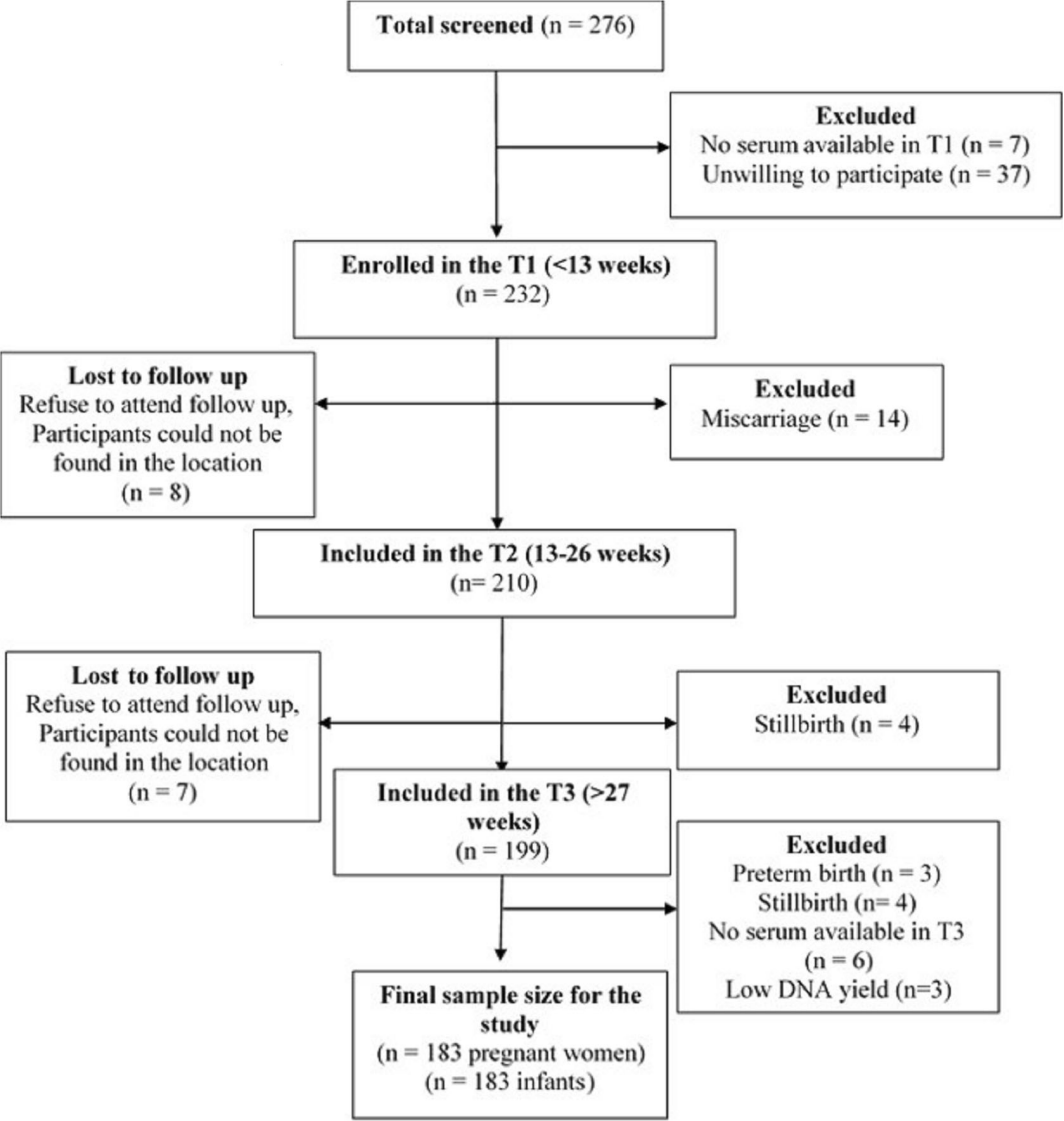
Flowchart showing the selection of study participants. Pregnant women who were < 13 weeks of gestation were recruited and followed up until the delivery to determine newborn anthropometry measurements. Out of 276 women, 90 were dropped out because of pregnancy loss, change of residence, not willing to continue research, and those who could not be contacted again. Out of 186 pregnant women who completed all requirements and attended follow-ups from the T1 to delivery, three individuals were excluded due to low DNA yield and hence a total of 183 mother and infant pairs were used for analysis. T1: first trimester; T2: second trimester; T3: third trimester

All participants were pregnant women who were recruited during their first antenatal care checks at the public health centres. Inclusion criteria included: 1) pregnant women willing to visit public health care at each site, 2) those who were in the T1 (<13 weeks) of their singleton pregnancy, 3) those who were healthy based on medical examination, and 4) those who were willing to participate by signing the informed consent and following the research procedures. Stratified random sampling was applied for the data collection that took place at two research locations: mountainous and coastal areas. Public health centers that had high numbers of the first-trimester pregnant mothers were chosen for the data collection. Women were excluded from the study if they had multiple pregnancies, some common complications of pregnancy such as preeclampsia, miscarriage or pregnancy loss, stillbirth, and they had chronic illness like diabetes, hypertension, cardiovascular disease, or hypothyroidism. Women who were taking drugs that can interfere with vitamin D metabolism such as antiepileptic agents, glucocorticoids, anti-oestrogens or antiretroviral drugs during pregnancy were excluded. Out of 239 women, 53 were dropped out for different reasons, including pregnancy loss, change of residence, not willing to continue research, and those who could not be contacted again. The number of pregnancy loss due to complications of pregnancy such as foetal inflammation, stillbirth, and abnormal foetal development was 25 (13.44%). There were 3 cases of preterm birth, 8 cases of stillbirth, and 14 cases of miscarriage. Finally, we obtained 186 pregnant women who completed all requirements and attended follow-ups from T1 to delivery. After excluding three samples due to low DNA yield, a total of 183 mother and infant pairs were used for the present study. Participant’s recruitment process is shown in detail in Fig. 1.

### Study Participant’s characteristics

Maternal sociodemographic factors were assessed using a standardized questionnaire administered by trained field data collector (enumerator, i.e., a registered nutritionist). The questionnaire included information on demographics, maternal occupation, education, and pregnancy profile. These data were prospectively collected from medical records or interviews. Maternal sociodemographic characteristics included age, education level (primary, secondary, and tertiary levels), maternal working status (working and not working), and geographical status (mountainous and coastal area). Maternal health status included pre-pregnancy BMI, and mid-upper arm circumference (MUAC). Maternal lifestyle included the outdoor activity to measure the sun exposure status during pregnancy and maternal vitamin D and calcium supplementation during pregnancy.

### Anthropometric measurements

Maternal anthropometric measurements (height, weight, and MUAC) were determined at enrolment and followed up during pregnancy. Pre-pregnancy BMI was calculated based on the height routinely measured at the clinic visit and pre-pregnancy body weight obtained at interview through maternal and child monitoring book. Maternal body weight was measured to the nearest 100 g using an electronic scale (Seca 815, Seca GmbH. Co. kg, Germany) and height was measured to the nearest millimeter using a stadiometer (Seca 217, Seca GmbH. Co. kg, Germany). The BMI calculation was based on the body weight (kg) divided by the square of body height (m). Pre-pregnancy BMI was classified according to World Health Organization guidelines for Asian populations (underweight, <18.5 kg/m^2^; normal, 18.5–23.49 kg/m^2^; overweight, 23.5–24.99 kg/m^2^; Pre-obese, 25–29.99 kg/m^2^; Obese, ≥30 kg/m^2^) [18].

### Measuring serum 25-hydroxyvitamin D levels

Maternal blood was collected two times under non-fasting conditions at <13 and > 27 weeks of gestation. Serum samples were stored at -70 °C until they were analyzed for 25(OH)D concentrations. Serum levels of 25(OH)D were assessed using Enzyme-linked immunosorbent assay (ELISA) from Diagnostic Biochemistry Canada (DBC) 25-Hydroxyvitamin D ELISA kit (DBC, London, Ontario Canada) and measured using xMark Microplate Spectrophotometer (Bio-Rad Laboratories Inc., Hercules, California, USA). The assay had a sensitivity of 5.5 ng/mL and an intra and inter-assay coefficient of variation of 5% and 8.1%, respectively. The vitamin D status was defined as serum 25(OH)D < 12 ng/mL (vitamin D deficient), 12–19 ng/mL (vitamin D insufficient), ≥20 ng/mL (vitamin D sufficient) according to Institute of Medicine (IOM) guidelines [19].

### SNP selection and genetic analysis

We selected six candidate SNPs according to the following criteria: (1) biological importance in vitamin D synthesis, metabolism, transportation, or degradation; (2) SNPs with minor allele frequency of >5%, and (3) evidence of a significant association in previous GWASs. The selected genes were *DHCR7* (rs12785878), *CYP2R1* (rs12794714), *GC* (rs2282679), *CYP24A1* (rs6013897), and *VDR* (rs2228570 and rs7975232) [12, 17, 20] and the roles of these genes in the vitamin D cascade are shown in Supplementary Fig. 1.

Blood samples were collected from all the study participants. Genomic DNA was isolated from peripheral blood leukocytes using PureLink Genomic DNA Mini Kit (Invitrogen, Carlsbad, USA). The DNA concentration was determined using a NanoDrop spectrophotometer (Isogen Life Science, De Meern, the Netherlands). Genotyping was performed at LGC Genomics, UK (http://www.lgcgroup.com/services/genotyping). Genotype frequencies were tested against the Hardy-Weinberg equilibrium (HWE) using the χ^2^ test. Genotype frequencies of all SNPs were in Hardy Weinberg equilibrium and the minor-allele frequencies of the SNPs ranged from 0.18 to 0.39 (Supplementary Table 1).

### Pregnancy outcomes

Gestational age at birth was calculated from estimated gestational age examined by obstetricians or midwives using transabdominal ultrasound performed or date of last menstrual period in the absence of ultrasound at the Maternal Clinic or Hospital. Infants’ birth weight, birth length, and head circumference were recorded at birth using Seca mechanical measuring scales (Seca 803, Seca GmbH. Co. kg, Hamburg, Germany). We classified newborn anthropometry status according to World Health Organization Child Growth Standards for head circumference-for-age (small head circumference, <35 cm and normal head circumference, ≥35 cm), weight-for-age (low birth weight, <2500 g and normal birth weight ≥ 2500 g), and length-for-age (short birth length, <50 cm and normal birth length, ≥50 cm) [21].

### Sample size and power calculation

The sample size was calculated for investigating the association between vitamin D levels and birth weight, which was the main objective of the VDPM study. Previous study found that 13.08 ng/mL difference of maternal vitamin D level between mothers of low birth weight neonate and those of normal birth weight neonate with standard deviation ranging from 18.50 to 20.16 ng/mL [22]. The sample size was calculated using the following formula [23].

$$ n=\frac{2{\left( Z\alpha + Z\beta \right)}^2{S}^2}{{\left(U1-U2\right)}^2} $$

n: Sample size of each group.
Zα: Value of standard normal distribution that is equal to α = 0.05 is 1.96.
Zβ: Value of standard normal distribution (90%) that equal to β = 0.10 is 1,28.
S: Outcome standard deviation based on the study by Khalessi et al. 2015 [23] is 18.5.
(U1-U2): Difference of mean outcome in low birthweight and normal birthweight status (13.08)
*n*: 2 (1,96 + 1,28)2 × 18.52/(13.08)2 = 41.96 ≈ 42.

Based on the above formula, the minimum number of samples required for each group is 42 to achieve a statistical power of 90% to test for the association between vitamin D levels and birth weight. Hence, we aimed to recruit a total sample size with minimum of 100 participants to account for a 20% drop-out. Given that there are no studies, to date, that have examined the association between genetic variants and vitamin D levels and adverse pregnancy outcomes in Indonesia, we were unable to calculate the power for the genetic analysis. Furthermore, genetic analysis was conducted as a retrospective post hoc analysis and hence the power calculation was not performed for the genetic study.

### Statistical analysis

Data were analysed using the IBM SPSS Statistics for Windows (version 23.0; SPSS, Inc., Chicago, IL, USA). Continuous variables with normal distribution were presented as mean ± SD. Categorical variables were presented as frequency and percentage. The normality of distribution of outcome variables (maternal serum 25(OH)D levels) was tested by Kolmogorov-Smirnov test.

Bivariate Pearson correlation was established to examine the correlation of serum 25(OH)D levels in the first trimester with serum 25(OH)D levels in the third trimester. A multinomial logistic regression model was used to identify the association between vitamin D status during pregnancy and newborn anthropometry status such as birth weight status, head circumference status, and birth length status. A multivariate analysis using general linear model (GLM) was conducted to determine the association between vitamin D status and newborn anthropometry. Significant factors associated with vitamin D status were entered into the GLM to adjust for covariate variables such as age, pre-pregnancy BMI, gestational age birth, infant gender, and supplement intake during pregnancy.

Genetic risk score (GRS), which was the sum of risk alleles from the SNPs rs12785878 (*DHCR7*), rs12794714 (*CYP2R1*), rs2282679 (*GC*), rs6013897 (*CYP24A1*), and rs2228570 and rs7975232 (*VDR*) [12, 17, 20], was created. Furthermore, GRS was divided into three groups as “vitamin D-GRS”, “synthesis-GRS” and “metabolism-GRS”. “Vitamin D-GRS” was obtained from all the six SNPs that play a role in the synthesis and metabolism of vitamin D. Two SNPs in genes encoding proteins involved in 25(OH)D synthesis (*DHCR7* and *CYP2R1*) were included in the “synthesis-GRS” [12] and four SNPs in genes encoding proteins involved in 25(OH)D metabolism (*GC*, *CYP24A1*, *VDR*) were included in the “metabolism-GRS” [20].

**Fig. 2:**
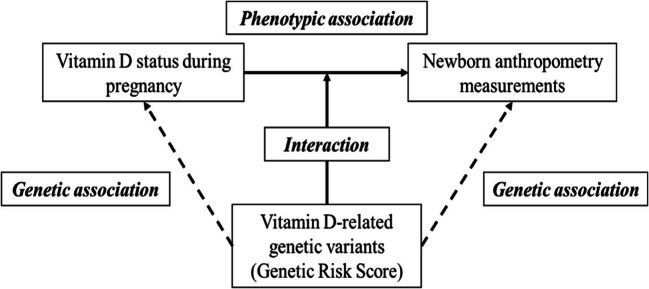
Diagram representing the study objectives**.** Three possible associations and one possible interaction were examined. Broken lines represent genetic associations and unbroken lines represent phenotypic association and interaction between genetic risk score (GRS) and vitamin D status on newborn anthropometry measurements, respectively. Phenotypic association between vitamin D status and newborn anthropometry measurements and the genetic associations between GRS and vitamin D status and newborn anthropometry measurements were investigated

The effect of GRSs on 25(OH)D levels and newborn anthropometry was assessed using univariate general linear models after adjustment for potential confounders (age, pre-pregnancy BMI, geography status, vitamin D and calcium supplement consumption during pregnancy and sunlight exposure status). The associations of GRSs with vitamin D status and newborn anthropometry (birth weight, birth length, head circumferences) were analysed using logistic regression analysis. The interaction between GRS and 25(OH)D levels during pregnancy (T1 and T3) on newborn anthropometry measurements was determined by including interaction terms [GRS*25(OH)D] in the model and adjusting for age, pre-pregnancy BMI, gestational age at birth, and infant’s gender. The study objectives are shown in Fig. 2.

Correction for multiple testing was performed using Bonferroni correction. Corrected *P* value for association analysis was ≤0.006 [3 GRS * 3 maternal 25(OH)D level outcomes (T1, T3, and changes in 25(OH)D during pregnancy) = 9 tests]. For the interaction analysis, corrected P value was ≤0.003 [3 GRS * 2 maternal 25(OH)D outcomes (T1 and T3) * 3 newborn anthropometry outcomes (birth weight, birth length, and head circumference) = 18 tests].

## Results

### Characteristic of the study population

The characteristics of the study participants stratified based on maternal vitamin D status at T1 and T3 are shown in Table 1**.** There was a significant difference in diastolic blood pressure (DBP), and body weight during the third trimester and there was a significant difference in outdoor activity (hours/day) during the first trimester between those who were vitamin D deficient (VDD) and those with normal vitamin D status (NVD) (*p* < 0.05). In Table 1, there was a significant difference in systolic blood pressure, bodyweight, and MUAC between T1 and T3 (p < 0.05, for all comparisons). Systolic blood pressure, bodyweight, and MUAC were significantly higher in T3 compared to T1. However, there was no significant difference in the levels of hemoglobin and diastolic blood pressure (*p* > 0.05, for all comparisons). The study participants were enrolled at an average age of 29.7 ± 5.68 years. The average of pre-pregnancy Body Mass Index (BMI) was 23.45 ± 4.56 kg/m^2^. The average gestational duration was 38.88 ± 1.91 weeks and 73.30% of deliveries were normal. Mean birth weight, birth length, and head circumference were 3204.87 ± 494.99 g, 48.56 ± 2.87 cm, and 33.89 ± 2.52 cm, respectively. Approximately 6.80% (*n* = 12) of newborn babies had low birth weight (LBW) status, while 5.40% (*n* = 10) were diagnosed with macrosomia. There were < 10% of cases who had adverse pregnancy outcomes such as LBW, SGA, and preterm birth (PTB). However, a higher number of women had babies with a small head circumference (<35 cm) and short birth length (<50 cm) (57.30% and 64.10%, respectively).

**Table 1:** Characteristics of study participants

Variables	T1					T3					T1		T3		
	n	VDD Status	n	NVD Status	P	n	VDD Status	n	NVD Status	P	n	Mean ± SD	n	Mean ± SD	P
Age, years	192	29.60 ± 5.51	40	30.53 ± 6.48	0.412	87	29.05 ± 5.21	99	30.36 ± 6.13	0.122					
Systolic, mmHg	192	110.94 ± 11.16	40	107.75 ± 11.87	0.124	87	111.36 ± 10.88	99	111.44 ± 9.79	0.962	186	107.08 ± 10.84	186	111.51 ± 10.26	**0.005**
Diastolic, mmHg	192	75.57 ± 7.08	40	74.63 ± 7.28	0.455	87	77.62 ± 8.61	99	75.37 ± 6.80	**0.047**	186	72.86 ± 7.36	186	76.60 ± 7.88	0.553
GA, weeks	192	9.67 ± 2.32	40	9.48 ± 2.53	0.661	87	30.49 ± 3.18	99	30.15 ± 2.93	0.442					
Hb, g/dL	192	11.62 ± 1.39	40	10.34 ± 1.15	0.180	87	10.82 ± 1.51	99	11.19 ± 1.50	0.650	186	11.58 ± 1.39	186	11.81 ± 1.36	0.413
Height, cm	192	154.51 ± 5.91	40	153.59 ± 6.47	0.409	87	154.68 ± 5.78	99	153.78 ± 6.65	0.228					
Bodyweight, Kg	192	56.61 ± 11.68	40	54.81 ± 11.70	0.380	87	63.67 ± 11.58	98	64.21 ± 10.71	**0.016**	186	56.32 ± 11.63	186	63.93 ± 11.15	**0.001**
BMI, kg/m^2^	192	23.54 ± 4.37	40	23.04 ± 5.42	0.590	87	23.23 ± 4.56	99	23.77 ± 4.56	0.842					
MUAC, cm	192	27.04 ± 3.78	40	26.81 ± 3.94	0.740	87	24.65 ± 3.66	99	27.90 ± 3.82	0.994	186	27.02 ± 3.81	186	27.82 ± 3.80	**0.001**
Outdoor activity, hours/day	192	59.22 ± 51.90	40	73.88 ± 38.02	**0.042**	87	60.35 ± 48.65	99	64.31 ± 54.51	0.604					
Birth weight, g						86	3244.90 ± 469.51	98	3147.09 ± 458.73	0.155					
Birth length, cm						86	48.59 ± 3.43	98	48.53 ± 3.05	0.890					
Head circumference, cm						86	34.10 ± 2.98	98	33.55 ± 1.89	0.128					
GA at birth, weeks						86	39.08 ± 1.81	98	38.73 ± 1.94	0.209					

*VDD* vitamin D deficient, *NVD* normal vitamin D, *GA* gestational age, *BMI* body mass index, *25(OH)D* 25-hydroxyvitamin D, *T1* first trimester, *T3* third trimester, *MUAC* mid-upper arm circumference. Data provided are mean ± standard deviation. Bold number presented as *P* < 0.05

Vitamin D status during pregnancy

Average maternal serum 25(OH)D level in T1 was 14.00 ± 6.97 ng/mL. Approximately 82.80% (*n* = 154) of women were deficient (47.30%, *n* = 88) and insufficient (35.50%, *n* = 66) for vitamin D. The serum 25(OH)D levels increased significantly during pregnancy (*P* = 0.0001, R = 0.425). In the T3, average maternal serum 25(OH)D level was 21.21 ± 10.16 ng/mL. A total of 46.80% (*n* = 87) of women were vitamin D sufficient, 34.40% (*n* = 64) were insufficient and 18.80% (*n* = 35) were deficient. The prevalence of vitamin D deficiency and insufficiency in the T1 lowered from 82.80% (*n* = 154) to 53.20% (*n* = 99) in the T3.

### Association between maternal Vitamin D status during pregnancy and newborn anthropometry

We found no significant association between 25(OH)D level during T1 and T3 and newborn anthropometric measurements (*P* > 0.05 for all comparisons). There was also no significant association between changes in vitamin D status during pregnancy and newborn anthropometry (P > 0.05 for all comparisons) **(**Table 2**)**.

**Table 2:** Association between Vitamin D Status during Pregnancy and Newborn Anthropometry

Variables	Newborn Anthropometries		
	Birth weight (g)	Birth length (g)	Head circumference (cm)
Sufficiency (*n* = 86)	3147.09 ± 458.73	48.53 ± 2.87	33.55 ± 1.89
Insufficiency (*n* = 63)	3246.03 ± 403.14	48.86 ± 1.89	34.21 ± 1.98
Defficiency (n = 35)	3242.86 ± 576.65	48.11 ± 5.17	33.91 ± 4.25
P value	0.301	0.618	0.386

Vitamin D status during pregnancy defined based on Institute of Medicine (IOM): sufficient (≥20 ng/mL), insufficient (12–19.99 ng/mL), and deficient (<12 ng/mL) [17]

*P* values were adjusted for age, pre-pregnancy BMI, preterm status, vitamin D intake, sun exposure status and consumption of vitamin D and calcium supplements

### Association between GRS and serum 25(OH)D levels during pregnancy

**Fig. 3:**
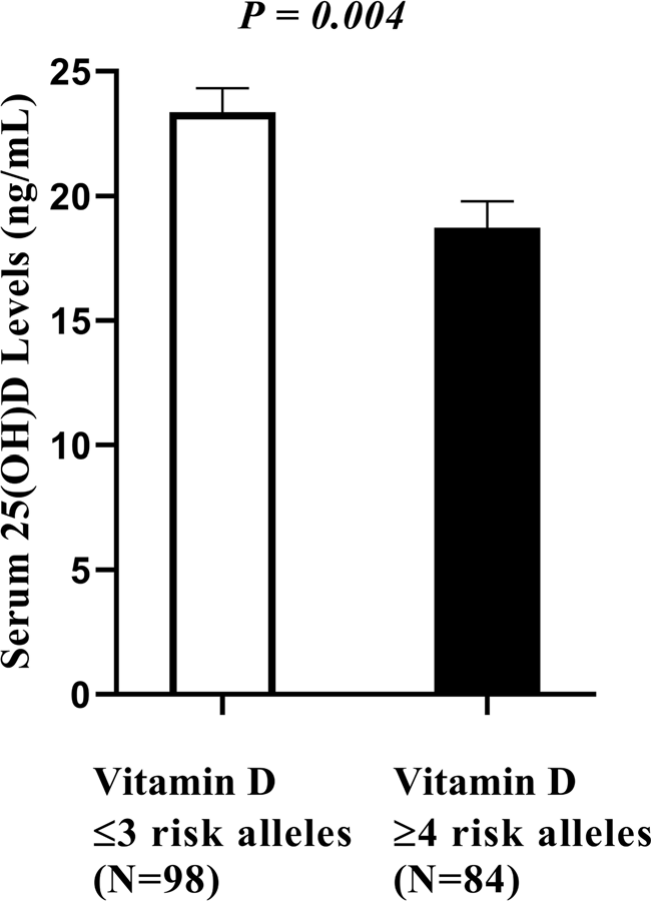
Association between vitamin D-GRS and serum 25(OH)D levels in T3. Among those who carried ≥4 risk alleles had lower serum 25(OH)D levels in T3 compared to women with ≤3 risk alleles (*P* = 0.004)

There was a significant association between vitamin D-GRS and 25(OH)D levels in T3 (P = 0.004) and changes in 25(OH)D levels during pregnancy (*P* = 0.018), but not with T1 25(OH)D levels (*P* = 0.157). The synthesis-GRS and metabolism-GRS had no effect on 25(OH)D levels and changes in 25(OH)D levels during pregnancy (*P* > 0.05 for all comparisons). The association between GRSs and serum 25(OH)D levels during pregnancy are shown in Table 3 and Fig. 3.

**Table 3:** Association pregnancy

Variables	25(OH)D T1 (ng/mL)		25(OH)D T3 (ng/mL)		Changes 25(OH)D (ng/mL)	
	Mean ± SD	P	Mean ± SD	P	Mean ± SD	P
Vitamin D-GRS total score*						
less than or equal 3 (n = 99)	14.77 ± 8.22	0.157	23.35 ± 10.65	0.004	8.58 ± 9.54	0.018
greater than or equal 4 (*n* = 85)	12.98 ± 5.40		18.74 ± 8.95		5.76 ± 9.50	
Synthesis GRS score**						
less than 2 (*n* = 137)	14.37 ± 7.65	0.182	21.80 ± 10.46	0.287	7.43 ± 9.64	0.724
greater than or equal 2 (*n* = 46)	12.72 ± 5.03		19.65 ± 9.06		6.93 ± 9.62	
Metabolism GRS score***						
less than or equal 3 (*n* = 147)	14.07 ± 7.55	0.655	21.63 ± 10.45	0.482	6.57 ± 9.53	0.643
greater than or equal 4 (*n* = 37)	13.44 ± 4.96		19.56 ± 8.73		6.11 ± 9.90	

Bold number indicate *P* < 0.05; 25(OH)D, 25-Hydroxyvitamin D levels; T1, First trimester; T3, Third trimester

*P* values were adjusted for age, BMI, vitamin D supplements, sun exposure status, and geographical status

*All six SNPs in genes involved in the synthesis and metabolism of vitamin D

**Two SNPs in genes encoding proteins involved in 25(OH)D synthesis (*DHCR7 and CYP2R1*) included in the “Synthesis score”

***Four SNPs in genes encoding proteins involved in 25(OH)D metabolism (*GC, CYP24A1, VDR*) are included in the “Metabolism score”

### Association between GRSs and newborn anthropometry

We observed no statistically significant association of the vitamin D-GRS, synthesis-GRS, and metabolism-GRS with newborn anthropometry measurements (*P* > 0.05 for all comparisons). Similar finding was observed even after classifying newborn anthropometry measurements into categorical variables (P > 0.05 for all comparisons) (Supplementary Tables 2 and 3).

### Interaction between GRS and 25(OH)D during pregnancy on newborn anthropometry

**Fig. 4:**
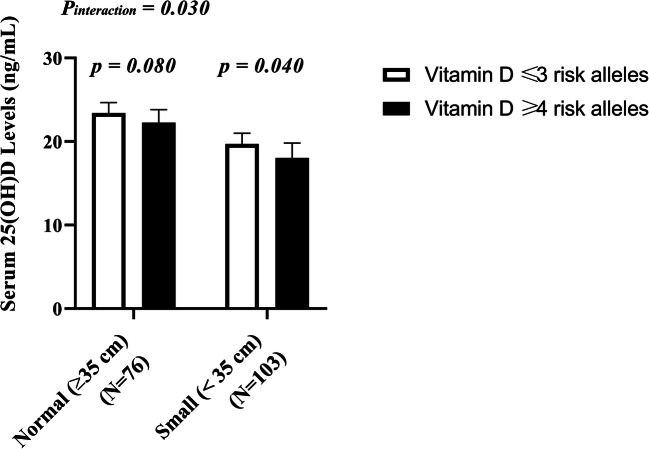
Interaction between vitamin D-GRS and 25(OH)D levels in T3 (ng/mL) on Head circumference. Mothers of neonates with head circumference < 35 cm had significantly lower 25(OH)D levels if they carried ≥4 risk alleles compared to those who carried ≤3 risk alleles (*P* = 0.040)

None of the interactions were statistically significant except for the interaction between vitamin D-GRS and 25(OH)D concentrations in T3 on newborn head circumference measurement (*P* = 0.030). Further stratification of study participants based on head circumference cut-off points (small heads, <35 cm and normal head, ≥35 cm) [18] showed that mothers of neonates with head circumference < 35 cm had significantly lower 25(OH)D levels if they carried ≥4 risk alleles compared to those who carried ≤3 risk alleles **(**Fig. 4**)**. However, after correction for multiple testing, this interaction was not considered statistically significant **(**Table 4**)**.

**Table 4:** Interaction between GRS and 25(OH)D on Newborn Anthropometry

*Interaction between the GRS and 25(OH)D T1 on newborn anthropometry measurements*		
Interaction between vitamin D-GRS*25(OH)D T1 on birth weight	Interaction between vitamin D-GRS*25(OH)D T1 on birth length	Interaction between vitamin D-GRS*25(OH)D T1 on head circumference
2.72 ± 10.55 (0.797)	0.04 ± 0.06 (0.510)	0.09 ± 0.05 (0.098)
Interaction between synthesis-GRS*25(OH)D T1 on birth weight	Interaction between synthesis-GRS*25(OH)D T1 on birth length	Interaction between synthesis-GRS*25(OH)D T1 on head circumference
−0.23 ± 14.19 (0.472)	−0.11 ± 0.08 (0.897)	0.07 ± 0.07 (0.312)
Interaction between metabolism-GRS*25(OH)D T1 on birth weight	Interaction between metabolism-GRS*25(OH)D T1 on birth length	Interaction between metabolism-GRS*25(OH)D T1 on head circumference
−5.31 ± 15.85 (0.738)	0.121 ± 0.10 (0.214)	0.02 ± 0.08 (0.799)
*Interaction between the GRS and 25(OH)D T3 on newborn anthropometry measurements*		
Interaction between vitamin D-GRS*25(OH)D T3 on birth weight	Interaction between vitamin D-GRS*25(OH)D T3 on birth length	Interaction between vitamin D-GRS*25(OH)D T3 on head circumference
9.56 ± 6.80 (0.162)	0.06 ± 0.04 (0.199)	0.08 ± 0.03 (0.031)
Interaction between synthesis-GRS*25(OH)D T3 on birth weight	Interaction between synthesis-GRS*25(OH)D T3 on birth length	Interaction between synthesis-GRS*25(OH)D T3 on head circumference
7.39 ± 8.14 (0.366)	0.04 ± 0.05 (0.426)	0.08 ± 0.04 (0.075)
Interaction between metabolism-GRS*25(OH)D T3 on birth weight	Interaction between metabolism-GRS*25(OH)D T3 on birth length	Interaction between metabolism-GRS*25(OH)D T3 on head circumference
5.99 ± 9.16 (0.514)	0.04 ± 0.056 (0.475)	0.08 ± 0.05 (0.105)

*T1* first trimester, *T3* third trimester, *25(OH)D* 25-hydroxyvitamin D

Values are beta coefficients ±standard errors. P values are provided within brackets

*P* values were adjusted for age, pre-pregnancy BMI, supplement consumption, gestational age at birth, and gender of the infants

### Association between SNPs and 25(OH)D during pregnancy

Besides exploring the impact of GRS on 25(OH)D levels during pregnancy, the individual effect of the SNPs on 25(OH)D levels was also examined. Under a dominant genetic model, *ApaI* (rs7975232) SNP showed a significant association with 25(OH)D levels in both T1 (0.047) and T3 (*p* = 0.043), where A allele carriers had significantly lower 25(OH)D concentrations. In addition, A allele carriers of the *CYP2R1* (rs12794714) SNP had significantly lower levels of 25(OH)D in both T1 (*p* = 0.001) and T3 (p < =0.0001). There was also a significant association between *GC* (rs22282679) SNP and 25(OH)D concentrations in T3 and changes in 25(OH)D levels during pregnancy (*P* < 0.001), but not in T1 (*P* = 081). None of the other associations were statistically significant (Supplementary Table 4).

## Discussion

To our knowledge, this is the first study of its kind to investigate whether maternal vitamin D status was associated with newborn anthropometry measurements using a genetic approach. Our study demonstrated a high prevalence (82.80%) of vitamin D deficiency among Indonesian pregnant mothers. Women who had ≥4 vitamin D-decreasing risk alleles had significantly lower levels of serum 25(OH)D during pregnancy. Even though there was no direct association between GRS and newborn anthropometric measurements, mothers of neonates with head circumference < 35 cm had significantly lower 25(OH)D levels if they carried ≥4 risk alleles suggesting that vitamin D deficiency during pregnancy can increase the genetic risk of adverse newborn anthropometry outcomes. Considering that more than half of the study participants were vitamin D deficient (83%), establishing a vitamin D prevention program for pregnant women may be considered to maintain optimal foetal growth and development. Our findings, if replicated in future studies, may have a significant public health impact on initiating strategy to raise the awareness on the importance of vitamin D during pregnancy to prevent vitamin D deficiency and its adverse pregnancy outcomes.

Recent studies have shown a significant phenotypic association between serum 25(OH)D levels during pregnancy and adverse pregnancy outcomes such as gestational diabetes mellitus, pre-eclampsia, SGA, LBW and PTB [22, 24–26]. Evidence from observational studies have suggested that lower maternal 25(OH)D concentrations are associated with LBW [7, 27, 28]. A recent prospective cohort study in 3658 Chinese mother-and-singleton-offspring pairs demonstrated that vitamin D deficiency during pregnancy was associated with neonatal birth size and estimated to double the risk of LBW [28]. In addition, two other studies that examined serum 25(OH)D levels during pregnancy found no association between first trimester vitamin D status and neonatal length but found a significant association in the third trimester [29, 30]. However, a few studies failed to show an association between maternal 25(OH)D levels and adverse pregnancy outcomes [29, 31–33]. These inconsistencies in findings could be due to confounding by unknown factors and the differences in cut-points of vitamin D status used, sample size, population characteristics, skin pigmentation, exposure to sunlight, vitamin D supplementation and methods to measure 25(OH)D [24–26, 29, 31–35]. Given these limitations, we used a genetic approach, which is less prone to confounding, to explore the association between serum 25(OH)D levels during pregnancy and adverse pregnancy outcomes.

One of the main findings of our study was the significant association between GRS (≥4 risk alleles) and lower serum 25(OH)D levels in the third trimester (*P* = 0.004) and changes in serum 25(OH)D levels during pregnancy. Our finding was similar to a study in 759 Chinese Han pregnant women from Zhoushan Pregnant Women Cohort (ZPWC) which also showed that individuals with >3 risk alleles had significantly lower 25(OH)D levels compared to those with 1 risk allele [36]. These findings are suggestive of the fact that the vitamin D-related genetic variants might have additive or synergistic effects in influencing 25(OH)D concentrations in pregnant mothers.

Very few studies have assessed the association of vitamin D-related genotypes with 25(OH)D and newborn anthropometry (birth weight, birth length, head circumferences). A few recent studies have shown that *VDR* gene variants influence birth weight and risk for SGA in black and white women [7, 27]. A recent Mendelian randomization study has also shown that polymorphisms in vitamin D-related genes, *CYP2R1* [rs10741657] and *DHCR7* [rs12785878], were associated with LBW suggesting a causal link between maternal vitamin D deficiency and neonatal birth weight [37]. Conversely, our study found no association between GRS and newborn anthropometry measurements (birth weight, birth length, head circumferences); however, mothers of neonates with small head circumference group (<35 cm) had significantly lower 25(OH)D levels if they carried ≥4 risk alleles suggesting that vitamin D deficiency could increase the genetic risk of adverse neonatal outcomes. Our finding is in line with a previous study which had also shown that mothers of neonates with small head circumference (<35 cm) had significantly lower levels of 25(OH)D [22**];** but the previous study did not explore the genetic susceptibility of the pregnant mothers. Future studies investigating the genetic basis of the associations between vitamin D status during pregnancy and newborn anthropometry measurements are required to confirm or refute our findings.

While most of the genetic variants chosen for our study have not been studied previously in relation to the risk of adverse pregnancy outcomes, *VDR* gene variants (rs2228570 and rs7975232) have been shown to be associated with the risk of adverse pregnancy outcomes such as PTB, LBW, and SGA status [27, 38–42]. However, there are also a few studies which failed to provide evidence for the relationship between rs7975232 (*VDR)* and PTB risk [38, 39]. We were unable to explore the association between *VDR* variants and PTB risk in the present study as the PTB variable was not available for all study participants; however, we examined other newborn anthropometry measurements such as birth weight, birth length and head circumference. *VDR* is required for the vitamin D metabolic pathway where its activation regulates the expression of genes involved in cell proliferation and differentiation [43]. Studies have shown the expression of *VDR* in placental tissues suggesting the role of vitamin D in reproduction and maternal to foetal nutrient transfer mechanism [44, 45]. Hence, the beneficial effects of vitamin D on foetal transfer mechanism can be affected by the decrease in *VDR* expression. Furthermore, it is possible that *VDR* might be a key factor in maternal to foetal nutrient transfer mechanism and adverse pregnancy outcomes and therefore serves as a strong candidate gene for our study.

The current study has some limitations. Firstly, the sample size was relatively modest; however, we were still able to identify significant associations and interactions in 183 mother and infant pairs after correction for multiple testing. Secondly, sunlight exposure variable was a self-reported outdoor activity and hence the bias involved in assessing sun exposure status cannot be ruled out. Thirdly, we have controlled for known major confounders, but we cannot completely exclude the possibility of other confounders such as the impact of vitamin D-fortified foods as this information was not collected in the present study. Compared to previous studies [1–3, 28], our study has several strengths. Firstly, the prospective cohort study analysis may reveal stable results and allows the examination of gestation-specific associations of maternal vitamin D status and newborn anthropometry. Secondly, measurements of 25(OH)D levels in different trimesters provides more information about the association between SNPs and vitamin D status during pregnancy. Fourthly, data were collected in the same season (dry season) and hence our study findings are unlikely to be affected by seasonal variation Thirdly, study participants were enrolled from single ethnicity (Indonesian Minangkabau women), which avoids genetic heterogeneity. Lastly, this is the first study of its kind in Indonesian pregnant mothers exploring the association of maternal vitamin D status and newborn anthropometry using a genetic approach which is less prone to confounding. Future research should focus on conducting large prospective studies, Mendelian Randomization studies and clinical trials to establish the causal effect of vitamin D deficiency on adverse pregnancy outcomes.

## Conclusion

In conclusion, we provide an evidence for an impact of vitamin D-related genetic variations on newborn anthropometry measurements through the influence of serum 25(OH)D levels among Indonesian pregnant Minangkabau women. Before initiating strategies for the implementation of vitamin D supplementation programs in Indonesia to prevent adverse pregnancy outcomes, further large studies are required to confirm our findings.

## Electronic supplementary material

ESM 1 (DOCX 214 kb)
