# Supplementary material for: A genetic approach to study the relationship between maternal Vitamin D status and newborn anthropometry measurements: the Vitamin D pregnant mother (VDPM) cohort study
Source: J Diabetes Metab Disord. 2020 Jan 27;19(1):91–103. doi: 10.1007/s40200-019-00480-5 (PMC7270445; doi:10.1007/s40200-019-00480-5)
Supplement: Supplementary file 1 — (DOCX 214 kb) [file 40200_2019_480_MOESM1_ESM.docx]

**Additional files**

**Supplementary Table 1: Genotype Distribution and Minor Allele Frequency (MAF)**

| Gene | n (%) | Minor Allele | N | Model | MAF | HWE |
| --- | --- | --- | --- | --- | --- | --- |
|  |  |  |  |  |  |  |
| *VDR* (rs2228570) |  | T | 180 | Dominant | 0.39 (T) | 0.053 |
| CC (common homozygotes) | 74 (41.10) |  |  |  |  |  |
| CT (heterozygotes) | 73 (40.50) |  |  |  |  |  |
| TT (rare homozygote) | 33 (18.30) |  |  |  |  |  |
| *VDR* (rs7975232) |  | A | 180 | Dominant | 0.33 (A) | 0.259 |
| CC (common homozygotes) | 78 (43.30) |  |  |  |  |  |
| AC (heterozygotes) | 86 (47.80) |  |  |  |  |  |
| AA (rare homozygotes) | 16 (8.90) |  |  |  |  |  |
| *DHCR7* (rs1278578) |  | T | 182 | Dominant | 0.24 (T) | 0.653 |
| GG (common homozygote) | 105 (57.70) |  |  |  |  |  |
| GT (heterozygotes) | 65 (35.70) |  |  |  |  |  |
| TT (rare homozygotes) | 12 (6.60) |  |  |  |  |  |
| *CYP2R1* (rs12794714) |  | A | 183 | Dominant | 0.28 (A) | 0.199 |
| GG (common homozygotes) | 98 (53.60) |  |  |  |  |  |
| AG (heterozygotes) | 67 (36.60) |  |  |  |  |  |
| AA (rare homozygotes) | 18 (9.80) |  |  |  |  |  |
| *GC* (rs2282679) |  | C | 184 | Dominant | 0.18 C) | 0.108 |
| AA (common homozygotes) | 119 (64.7) |  |  |  |  |  |
| AC (heterozygotes) | 62 (33.70) |  |  |  |  |  |
| CC (rare homozygotes) | 3 (1.60) |  |  |  |  |  |
| *CYP24A1* (rs6013897) |  | A | 183 | Dominant | 0.30 (A) | 0.328 |
| TT (common homozygotes) | 93 (50.80) |  |  |  |  |  |
| AT (heterozygotes) | 71 (38.80) |  |  |  |  |  |
| AA (rare homozygotes) | 19 (10.40) |  |  |  |  |  |

MAF, minor allele frequency; HWE, Hardy-Weinberg equilibrium.

**Supplementary Table 2: Association between GRS and Newborn Anthropometry Status as Categorical Outcomes**

| Outcomes | | Total GRS * | | | Synthesis GRS ** | | | Metabolism GRS *** | | |
| --- | --- | --- | --- | --- | --- | --- | --- | --- | --- | --- |
|  |  | **P** | **OR** | **95%CI** | **P** | **OR** | **95%CI** | **P** | **OR** | **95%CI** |
| Birth length | Normal |  |  |  |  |  |  |  |  |  |
|  | Short | 0.532 | 0.826 | 0.453-1.505 | 0.115 | 0.560 | 0.849-3.550 | 0.454 | 1.336 | 0.626-2.852 |
| Head circumferences | Normal |  |  |  |  |  |  |  |  |  |
|  | Small | 0.278 | 0.497 | 0.140-1.760 | 0.814 | 1.183 | 0.291-4.801 | 0.984 | 1.008 | 0.463-2.197 |
| Birthweight | Normal |  |  |  |  |  |  |  |  |  |
|  | Low | 0.584 | 1.426 | 0.401-5.075 | 0.504 | 1.639 | 0.385-6.965 | 0.841 | 1.186 | 0.225-6.254 |

OR, odds ratio; GRS: genetic risk score.

Binary logistic regression; Adjusted for age, BMI, infant gender, and gestational age.

*All six SNPs from the genes that are involved in synthesis and metabolism of vitamin D

**Two SNPs in genes encoding proteins involved in 25(OH)D synthesis (*DHCR7* and *CYP2R1*) included in the “Synthesis score”

***Four SNPs in genes encoding proteins involved in 25(OH)D metabolism (*GC, CYP24A1, VDR*) are included in the “Metabolism score”

**Supplementary Table 3: Association between GRS and Newborn Anthropometric Measurements as Continuous Outcomes**

| Outcomes | | GRS total score* | | Synthesis GRS score** | | Metabolism GRS score*** | |
| --- | --- | --- | --- | --- | --- | --- | --- |
|  |  | **≤ 3 risk alleles** | **≥ 4 risk alleles** | **< 2 risk alleles** | **≥2 risk alleles** | **≤ 3 risk alleles** | **≥ 4 risk alleles** |
| Birth weight | Mean±SD | 3176.53±501.96 | 3226.79±427.13 | 3190.07±468.99 | 3240±468.75 | 3201.71±486.42 | 3191.67±392.34 |
|  | p value | 0.494 | | 0.900 | | 0.796 | |
| Birth length | Mean±SD | 48.42±3.56 | 48.75±1.79 | 48.43±3.18 | 49.00±1.68 | 48.62±3.12 | 48.36±1.53 |
|  | p value | 0.405 | | 0.387 | | 0.542 | |
| Head circumference | Mean±SD | 33.76±3.04 | 33.95±1.83 | 33.80±2.73 | 33.93±1.93 | 33.85±2.70 | 33.83±1.84 |
|  | p value | 0.579 | | 0.788 | | 0.641 | |

Adjusted for age, BMI, gender of the infant, labour status, and gestational age.

* All six SNPs from the genes that are involved in synthesis and metabolism of vitamin D

**Two SNPs in genes encoding proteins involved in 25(OH)D synthesis (*DHCR7* and *CYP2R1*) included in the “Synthesis score”

***Four SNPs in genes encoding proteins involved in 25(OH)D metabolism (*GC, CYP24A1, VDR*) are included in the “Metabolism score”

**Supplementary Table 4: Association between Dominant Genetic Models and 25-Hydroxyvitamin D Levels during Pregnancy**

| Genes | 25(OH)D T1 | | 25(OH)D T3 | | Changes 25(OH)D (T3-T1) | |
| --- | --- | --- | --- | --- | --- | --- |
|  | **Mean±SE** | **P value** | **Mean±SE** | **P value** | **Mean±SE** | **P value** |
| *FokI* (rs2228570) |  |  |  |  |  |  |
| CC | 13.11±0.796 | 0.172 | 20.48±1.137 | 0.406 | 7.31±1.098 | 0.955 |
| C/T | 14.54±0.664 |  | 21.72±0.948 |  | 7.23±0.916 |  |
| *ApaI* (rs7975232) |  |  |  |  |  |  |
| CC | 15.12±0.779 | **0.047** | 22.91±1.100 | **0.043** | 7.88±1.070 | 0.452 |
| C/A | 13.03±0.679 |  | 19.91±0.960 |  | 6.80±0.934 |  |
| *DHCR7* (rs12785878) |  |  |  |  |  |  |
| GG | 13.66±0.669 | 0.519 | 20.59±0.945 | 0.333 | 6.93±0.914 | 0.593 |
| G/T | 14.33±0.782 |  | 22.01±1.105 |  | 7.68±1.069 |  |
| *CYP2R1* (rs12794714) |  |  |  |  |  |  |
| GG | 15.55±0.667 | **0.001** | 23.59±0.944 | **0.0001** | 8.06±0.941 | 0.242 |
| G/A | 12.12±0.717 |  | 18.57±1.013 |  | 6.44±1.010 |  |
| *GC* (rs22282679) |  |  |  |  |  |  |
| AA | 14.56±0.622 | 0.097 | 23.22±0.854 | **0.0001** | 8.63±0.841 | **0.008** |
| A/C | 12.80±0.844 |  | 17.55±1.160 |  | 4.79±1.141 |  |
| *CYP24A1* (rs6013897) |  |  |  |  |  |  |
| TT | 13.83±0.710 | 0.792 | 21.91±1.005 | 0.361 | 8.17±0.968 | 0.205 |
| T/A | 14.09±0.722 |  | 20.59±1.022 |  | 6.41±0.984 |  |

Linear regression model. Bold number is indicate P<0.05.

Adjusted for age, pre-pregnancy BMI, vitamin D supplements, sun exposure status, and geographical status

**
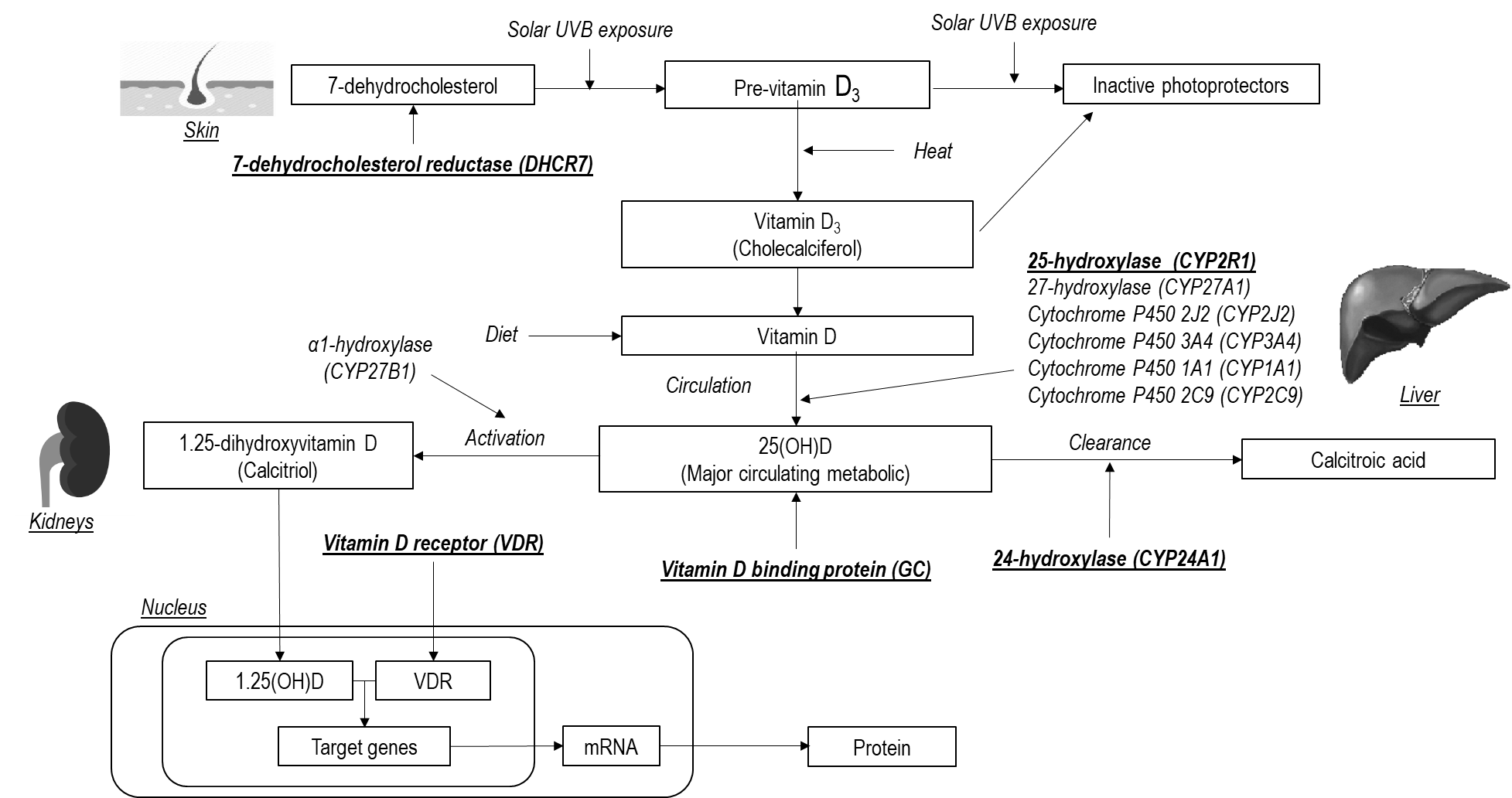
**

**Supplementary figure 1.** The selection of candidate genes in vitamin D synthesis and metabolic pathway. The main source of vitamin D in the body is obtained from exposure to UVB sunlight against the skin. Skin exposure to UVB radiation triggers the conversion of 7-dehydrocholesterol to pre-vitamin D_3_. *DHCR7* gene regulates the enzyme 7-dehydrocholesterol reductase, which converts 7-dehydrocholesterol to cholesterol by removing some substrates from the synthetic pathway of vitamin D3. The pre-vitamin D_3_ is converted to vitamin D3 through an heat-dependent process. Another form of vitamin D from food is vitamin D2, which along with vitamin D3, is transported to the liver, where it is converted by vitamin D-25- hydroxylase (*CYP2R1*) to 25-hydroxyvitamin D (25(OH)D). CYP2J2, CYP3A4, CYP27A1, CYP1A1, and CYP2C9 enzymes also contribute to the process. 25(OH)D is the main circulating form of vitamin D in the body and inactive, which is used to determine vitamin D status. Bound to vitamin D-binding protein (GC), 25(OH)D is transported to the kidneys and converted by 25-hydroxyvitamin D-1a - hydroxylase (1-OHase) (*CYP27B1*) to the biologically active form 1,25-dihydroxyvitamin D3 (Calcitriol). Calcitriol increases the expression of 24-hydroxylase (24-OHase) to catabolise 25(OH)D to the water-soluble, biologically inactive calcitroic acid, which is excreted in the bile. In the nucleus, 1,25(OH)D is bound by a vitamin D receptor (VDR) to perform a transcriptional function to gene target. *DHCR7* and *CYP2R1* function upstream of the production of 25(OH)D and hence, termed as 25(OH)D synthesis indicators, while *GC, CYP27B1 and CYP24A1* function downstream of the 25(OH)D production and hence, termed as 25(OH)D metabolism indicators.
